# 

**Published:** 1842-07-01

**Authors:** 


### MEDICO-CHIRURGICAL

### REVIEW,

AND

### JOURNAL

OF

### PRACTICAL MEDICINE.

*(NEW SERIES.)*

VOLUME THIRTY-SEVEN,

[1st of APRIL to 30th of SEPTEMBER,]

1842.

VOL. XVII. of DECENNIAL SERIES.

EDITED

BY JAMES JOHNSON, M.D.

PHYSICIAN EXTRAORDINARY TO THE LATE KING,

AND

HENRY JAMES JOHNSON, ESQ.

LECTURER ON ANATOMY AT THE SCHOOL OF ST. GEORGE'S HOSPITAL IN KINNERTON STREET.

Surgeon Gen'l's Office LIBRARY 5747 Washington, D.C.

LONDON:

PUBLISHED BY S. HIGHLEY, 32, FLEET STREET,

*Re-printed in New York, by Mr. Wood.*

1842.

WI
ME75T

PRINTED BY F. HAYDEN,
Little College Street, Westminster.

### INDEX.

A

Aberdeen, lunatic asylum of........ 256
Abscess of the uterus.............. 222
Abscess of the vagina ............ 227
Abscess, venous hæmorrhage from an 597
Abscesses, urinary................ 131
Abuse of corsets.................. 167
Accident to a diver............... 633
Action of plants on carbonic acid.... 163
Adulteration of the milk .......... 552
Advantages of early clinical study. .. 176
Affections of the spermatic cord .... 134
Aimé-Martin on education of mothers 113
Air, influence of, on the fœtus...... 484
Allnatt on tic douloureux.......... 151
Alopecia.......................... 397
Amaurosis ........................ 193
Ammonia, generation and influence of 162
Amputation of gangrened limbs .... 174
Analysis of the Springs of Homburg.. 504
Anatomy of the perineum, Monro's.. 157
Aneurysm of the pulmonary artery.. 3
Aneurysm, inguinal, treated by ligature 579
Aneurysm, aortic, burst into bronchus, 616
Aneurysms within the skull ........ 577
Animal substances, component materials of ....................... 161
Animal heat ...................... 163
Animal chemistry, Liebig's ........ 337
Another blow to Broussaism........ 165
Antiphlogistic medication.......... 106
Appendix, lodgement of a pin in the 5
Arachnitis, diffuse................ 61
Armies, purulent ophthalmia of .... 187
Arsenic, oxydes of iron in poisoning by, 208
Arteria hæmorrhoidalis inferior .... 160
Arteries, torsion of............... 172
Artery of the bulb, irregularity of the 159
Artery of the prostate ............ 160
Artificial pupil, formation of....... 191
Artificial inflation ............... 216
Artificial Harrowgate water ........ 621

B.

Babington's case of distended gall-bladder ...................... 243
Baker's case of cancer of the cranium 598
Barlow on disease of the kidney .... 209
Becquerel's therapeutics of children.. 104
Belfast lunatic asylum ............ 333
Bell on diabetes .................. 520
Bethlem, admission of pupils to .... 147
Bird on urinary concretions........ 232
Bladder, diseases of the neck of the.. 401
Bladder, neuralgia of the neck of the.. 401
Bladder, diseases of the body of the.. 417
Bladder, fungus and polypus of the.. 417
Bladder, cancer of the ............ 417
Bladder, tumors of the............ 418
Bladder, hernia of the ............ 419
Bladder, catarrh of the ............ 429
Blind fistulæ in perineo........... 440
Blistering plaister ............... 268
Blood, Orfila on the .............. 565
Bone, King on reparation of........ 461
Bougie, introduction of the ........ 438
Bougies, use of, in stricture ........ 122
Bouvier on myotomy............... 179
Brain, Foville on the.............. 1
Brain, functions of the............ 26
Brain, Lees on hypertrophy of the .. 608
Bright's disease after scarlatina .... 91
British Association, report to the.... 538
Brodie on the urinary organs ...... 401
Bronchial ganglionic phthisis ...... 466
Bronchus, aortic aneurysm in ...... 616
Broussaism, another blow to........ 165
Byron on malignant disease of the eye 251

C.

Calculi of the liver................ 12
Calculi in Guy's Hospital Museum .. 233
Cancer of the cranium ............ 598
Carbonic acid, action of plants on.... 163
Carcinoma uteri.................. 225
Carmichael on medical reform ...... 153
Cartilaginous disease, case of ...... 596
Cases and observations, surgical, Syme's 587
Catalepsy ........................ 557
Cataract ......................... 192
Cataract, Serre on the operation for 180
Catarrh of the bladder ............ 429
Cauda equina, tumor of the ........ 7
Caustic, use of, for stricture........ 124
Ceely on the variolæ vaccinæ ...... 13
Cerebrum and cerebellum, structure of the.......................... 28
Chelsea, military asylum, report from 289
Chemistry, organic, Dumas on.. 161, 534
Chemistry, Liebig's animal ........ 337
Chester Infirmary, report from...... 245
Children's Hospital, clinique of the.. 86
Children, pleurisy in .............. 93
Children, therapeutics of .......... 104
Children, hæmorrhages in ........ 108
China, medical missionary society in 149
China, ophthalmia in.............. 149
China, physic in.................. 262
China, state of the faculty in ...... 262
Chinese pathology ................ 263
Chirayta, infusion of.............. 265
Chusan, description of ............ 150

Citrate and ammonio-citrate of iron.. 621
Civiale on the genito-urinary organs 116
Civiale on the genito-urinary organs 401
Civilization, progress of .......... 37
Clark's letter on medical reform .... 153
Clay on the use of ox-gall.......... 278
Climate, influence of, on consumption 43
Climate of the United States ...... 135
Climate of Devonshire, Shapter's.... 491
Clinical study, advantages of........ 176
Clinical practice of M. Roux....... 584
Clinique of the Children's Hospital.. 86
Clinique des hopitaux des enfans .... 465
Cock on injuries of the head........ 229
Consciousness, conditions necessary for 18
Constitution, influence of, on surgical diseases ...................... 177
Consumption, statistics of ........ 41
Consumption, influence of localities on 41
Consumption, pathology of ........ 46
Consumption, prevention of ...... 51
Cornea, opacities of the............ 190
Corsets, use and abuse of.......... 167
Costello on fistula ................ 457
Counter-irritants ................ 276
Cranium, compound fracture of .... 581
Cranium, lardaceous cancer of the .. 598
Creosote, tincture of ............ 565
Creosote in encysted tumors........ 618
Criminal jurisprudence, Sampson on 79
Croup, laryngo-tracheotomy for .... 87
Curvature of the spine ............ 515
Cyclopædia of surgery ............ 457

D.

Dawlish ........................ 496
Deafness cured by morphia ........ 559
Death from high temperature ...... 139
Death from hæmorrhage of the lungs 287
Death of M. Double .............. 529
Death of the Duke of Orleans ...... 574
Decussation of the optic nerves...... 20
Detection of sugar in the urine...... 3
Devonshire, Shapter's climate of .... 491
Diabetes, Bell on.................. 520
Diabetes, with fatty matter ........ 522
Diarrhœa and dysentery............ 136
Diet in cases of severe wounds ...... 171
Diffuse cellular inflammation........ 60
Diffuse arachnitis ................ 61
Digestion, Dumas' theory of ........ 164
Disease, Gully's treatment of........ 497
Diseases of the scalp, Erichsen on.... 579
Dissection wounds, pathology of .... 60
Diver, accident to a................ 633
Dorton chalybeate ................ 286
Double, M. death of .............. 529
Downie on the springs of Homburg.. 503
Dropsy after scarlatina, iodine for.... 274
Druitt's surgeon's vade mecum...... 520
Dumas on organic chemistry.... 161, 534
Dumas' theory of digestion ........ 164
Dyspepsia, treatment of .......... 499

E.

East India command .............. 65
Eczema of the scalp .............. 385
Education of mothers, Aimé-Martin on 113
Education, &c. free-trade in ........ 619
Elements of pathology, Fletcher's.... 371
Encysted tumors of the vagina...... 228
Encysted tumors treated by creosote, 618
Enfans, clinique des hôpitaux des.... 465
Epidemic meningitis, history of an .. 541
Epilepsy, Jackson on.............. 110
Epilepsy, proximate cause of........ 111
Erichsen on diseases of the scalp .... 379
Erysipelas, Nunneley on............ 59
Erysipelas, treatment of............ 63
Exeter, medical topography of ...... 8
Exeter, diseases of................ 8
Extinction of human races.......... 259
Eye, mechanical lesion of the........ 186
Eye-ball, prominence of the ........ 197
Eyes, Serre on inflammation of the .. 180

F.

Facial neuralgia, Dr. Allnatt on...... 151
False passages in the urethra........ 129
Fermentative processes in living bodies 163
Fever, remedies for .............. 2
Fever, morbid anatomy of.......... 246
Fevers of India, Dr. Heyne on ...... 141
Fine words in medicine............ 268
Fisher on tumors of the cauda equina 7
Fistulæ, urinary ................ 133
Fistula lachrymalis................ 183
Fistula lachrymalis, Rognetta on.... 183
Fistulæ in perineo ................ 446
Fletcher's elements of pathology .... 371
Fœtus, influence of air on the ...... 484
Forry on the climate of United States 135
Fournet on pulmonary phthisis...... 68
Foville on the brain .............. 1
Fracture, Macdonnell on .......... 457
Fracture, compound, of cranium.... 581
France, vaccination in ............ 205
France's case of irideremia ........ 242
Free-trade in education & quackery.. 619
Functions of the grey and white matter 18
Functions of the brain ............ 26
Functions of the spleen, Jackson on.. 110

G.

Gall-bladder, case of distended...... 243
Gangrene, hospital, .............. 175
Gangrene produced by the starched bandage ...................... 600
Gangrened limbs, amputation of .... 174
Genital organs, affections of the, in children .................... 410
Genito-urinary organs, Civiale on, 116, 401

Germany, pharmacy in ............ 265
Gilbert on pulmonary consumption .. 41
Glandulæ aggregatæ ............... 247
Glover's, Dr. case of tubarian gestation 615
Gout, Weatherhead on ............ 509
Gout, hydropathic cure of..... .... 509
Great Britain, pharmacy in ........ 617
Green on a new mode of amputation 600
Gregory's Liebig's animal chemistry.. 337
Gully's treatment of disease ........ 497
Gunshot-wounds, treatment of...... 171
Guy's hospital reports ............ 209
Guy's hospital museum, calculi in .. 233

H.

Hæmaturia, case of................ 212
Hæmaturia......................... 426
Hæmorrhage from lithotomy........ 159
Hæmorrhage of the lungs, death from, 287
Hæmorrhages in children .......... 108
Hæmorrhoidal artery, inferior .. ... 160
Hallucinations and illusions ........ 526
Hanwell lunatic asylum............. 333
Harrison on deformities of the spine.. 515
Harrowgate water, artificial ........ 621
Hastings, Dr. Mackness on.......... 511
Head, cases of injury of the ........ 229
Head and face, malignant diseases of, 604
Hearing, metastasis of ............ 557
Heart, motions and sounds of the .. 2
Heart, movements and sounds of the, 538
Hernia, strangulated—operation .... 580
Hernia, strangulated ventral........ 582
Herpes circinnatus................. 389
Heyne on hill fevers .............. 141
High temperature, death from ...... 139
Hill fevers, Dr. Heyne on .......... 141
History of an epidemic meningitis.... 541
Homburg, Downie on the springs of.. 503
Hope, Dr. memoir of............... 53
Hôpitaux des Enfans, clinique des.... 465
Hospital gangrene ................ 175
Hôtel Dieu, surgical practice of the.. 560
Hughes on pulmonary phthisis....... 238
Human races, extinction of ........ 259
Hydrocele, iodine injections for...... 264
Hydropathic cure of gout........... 509
Hydrops pericardii, diagnosis of .... 3
Hypertrophy of the brain, Lees on... 608
Hysteria......................... 557

I.

Impetigo......................... 390
Incontinence of urine ........ 423, 450
India, diet in ................... 262
Indian army, Mr. Martin's note on the, 625
Induration of the os uteri.......... 221
Infanticide, Taylor on ............ 214
Infanticide, hydrostatic test for ..... 215
Infanticide, Ploucquet's test for ..... 215
Infants, purulent ophthalmia of ..... 188
Infants, on the management of...... 566
Infiltration of urine ............... 130
Infinity, sense of.................. 113
Inflammation of the eyes, M. Serre on, 182
Influence of constitution on surgical diseases ...................... 177
Influence of external circumstances on wounds ...................... 177
Infusion of chirayta .............. 265
Injury of the head, cases of ........ 229
Injury of the intestine, Key's case of.. 239
Insane, society for the relief of the .. 336
Insanity, Prichard on.............. 522
Internal pudic artery.............. 159
Intestine, injury of the ........... 239
Iodide of potassium in ophthalmic diseases ......................... 612
Iodine, effects of................. 4
Iodine injections for hydrocele ...... 264
Iodine for dropsy after scarlatina.... 274
Irideremia, France on ............ 242
Iron, oxydes of, in poisoning by arsenic 208
Iron, preparations of .............. 275
Iron, citrate and ammonio-citrate of.. 621
Irregularity of the pudic artery...... 159
Irregularity of the artery of the bulb.. 159

J.

Jackson on epilepsy .............. 110
Jackson on the functions of the spleen 110

K.

Kennion on perforation of the stomach 596
Key's case of injured intestine ...... 239
Kidney, Barlow on disease of the.... 209
King on reparation of bone ........ 461
Kingdon's case of disease of the appendix ........................ 5

L.

Lardaceous cancer of the cranium .. 597
Laryngo-tracheotomy for croup .... 87
Lateral curvature ....... ........ 515
Lawrance's medical report.......... 289
Lee on the education of mothers .... 113
Lees on hæmorrhage of the lungs.... 287
Lees on hypertrophy of the brain.... 608
Leg, amputation of the ............ 600
Lesion of sensibility and motion .... 586
Letter from Mr. Liddell............ 633
Lever on pelvic tumors obstructing parturition .................... 219
Liddell, Mr. letter from............ 633
Liebig's animal chemistry......... 337
Life and mind.................... 16
Ligature of ext. inguinal artery, &c... 579
Light from living human subjects ... 513
Lithotomy, operation for .......... 158
Lithotomy, hæmorrhage from ...... 159
Lithotomy, remarks on ............ 172
Lithotrity, Brodie on .............. 452

Lithotrity, dangers of.............. 455
Liver, calculi of the .............. 12
Location of pulmonary phthisis...... 238
London, mortality tables of ........ 277
Longet's vivisections .............. 536
Loudon on population ............... 501
Lumbar curvature.................... 520
Lunatic asylum of Aberdeen.......... 256
Lunatic asylum, Belfast............. 333
Lunatic asylum, Hanwell ............ 333
Lungs, absolute weight of the ...... 216
Lungs, diseases of the ............. 493
Lungs, penetrating wound of both .. 614

M.

Macdonnell on fracture.............. 457
Mackness on Hastings ............... 511
Madras Medical Journal ............. 137
Malignant diseases of the uterus .... 225
Malignant diseases of the vagina .... 228
Malignant disease of the eye-ball.... 251
Malignant diseases of the head and face 604
Management of infants .............. 566
Marsh on the evolution of light .... 513
Martin's Mr. note on the Indian army, 625
Mayo on the nervous system.......... 16
Meath Infirmary..................... 251
Mechanical lesions of the eye ...... 186
Medical topography of Exeter ...... 8
Medical Missionary Society in China, 149
Medical reform, Sir J. Clark on ..... 153
Medical reform, Dr. Carmichael on .. 153
Medicine, fine words in. ........... 268
Memoir of Dr. Hope ................. 53
Meningitis, history of an epidemic .. 541
Mesmero-phrenology ................. 593
Metastasis of hearing............... 557
Michel, Baron, on typhoid fever .... 165
Military asylum, report from the .... 289
Milk, state of the, during pregnancy.. 549
Milk, on the qualities of the ........ 550
Milk, formation of the ............. 551
Milk, adulteration of the .......... 552
Mind or matter ..................... 602
Missionary Society, medical, in China 149
Monomania........................... 525
Monro's anatomy of the perineum ... 157
Montgomery on pregnancy............ 336
Moral insanity ..................... 523
Morbid anatomy of................... 246
Morphia, deafness cured by ......... 559
Mortality tables of London ......... 277
Mucous inflammations, oxalic acid for 564
Muscular sense ..................... 24
Myotomy in spinal deformities...... 179

N.

Navy statistical reports ........... 65
Neck of the bladder, diseases of the.. 401
Neck of the bladder, neuralgia of the, 401
Neck, scrofulous ulcer of .......... 583
Nervous palpitation, diagnosis of .... 3
Nervous system, Mayo on the ....... 16
Nervous system, composition of the.. 17
Neuralgia, facial, Dr. Allnatt on.... 151
Neuralgia of the neck of the bladder.. 401
New York Hospital .................. 252
Nitrate of silver, discolouration by .. 610
Nose, operation for obliquity of the .. 201
Nunneley on erysipelas ............. 59

O.

Obliquity of the nose, operation for.. 201
Obstructions of the urethra, from injury ... 447
Ocular myotomy..................... 195
Opacities of the cornea............. 190
Operation for cataract, Serre on the.. 180
Operations en deux temps............ 171
Operations, mortality of ........... 250
Operations, statistics of........... 588
Ophthalmia in China ................ 149
Ophthalmia, purulent ............... 187
Ophthalmia, scrofulous.............. 188
Ophthalmia, syphilitic ............. 188
Ophthalmia, occasional forms of .... 190
Ophthalmic diseases, iodide of potas. in 612
Ophthalmological literature ........ 183
Optic nerves, decussation of the .... 20
Optic nerve, rupture of the ........ 184
Orbit, wound of the ................ 184
Orfila on the blood................. 565
Organic chemistry, Dumas on ..161, 534
Organic chemistry, Liebig's ........ 337
Orleans, death of the Duke of ...... 574
Os uteri, induration of the.......... 221
Os uteri, prolongation of the ....... 223
Oxalic acid for mucous inflammations 564
Ox-gall, Dr. Clay on ............... 278
Oxydes of iron as counter-poisons to arsenic.................... 208

P.

Pancreas, scirrhus of the .......... 274
Paralysis, sympathetic ............. 331
Parturition, pelvic tumors obstructing 219
Pathology, Fletcher's elements of.... 571
Patients, kindness towards.......... 178
Perception, laws of................. 36
Pelvic tumors obstructing parturition 219
Perforation of the stomach ........ 596
Perineum, Monro's anatomy of the .. 157
Peritonitis family disposition to .... 12
Pharmacy in Germany ............... 265
Pharmacy in Great Britain.......... 617
Phthisis, prevention of ............ 51
Phthisis, Fournet on ............... 68
Phthisis, hereditary transmission of.. 71
Phthisis, diagnostic signs of ........ 75
Phthisis pulmonalis, on the location of 238
Phthisis, bronchial ganglionic ...... 466
Phthisis, influence of tobacco on.... 541

Phthisis, Rayer on................ 562
Physic in China.................. 262
Physiological maxims.............. 568
Physiology of plants.............. 534
Pin in the appendix, case of........ 5
Pitting in small-pox, application for.. 3
Pityriasis....................... 397
Plants, action of, on carbonic acid.... 163
Pleurisy in children .............. 91
Pleurisy, causes of................ 94
Pleurisy, symptoms of ............ 96
Pleurisy, treatment of ............ 102
Ploucquet's test in infanticide ...... 215
Polypous tumors, nasal............ 252
Poor-law medical relief........... 269
Population, Loudon on............ 501
Pregnancy, urine a test of......... 4
Pregnancy and delivery, relations between ...................... 476
Pregnancy, state of the milk during.. 549
Pregnant women, diseases of........ 476
Pregnant women, regimen of........ 486
Preparations of iron .............. 275
Prichard on insanity .............. 522
Prostate, diseases of the .......... 412
Prostate, enlarged ................ 412
Prostate, suppuration of the........ 416
Prostate, atrophy of the .......... 416
Prostatic artery .................. 160
Prostatic veins ................... 160
Prostatic calculi.................. 417
Provincial Association, transactions of 1
Ptosis ........................ 185
Pudic artery, irregularity of the .... 159
Pulmonary artery, aneurysm of the.. 3
Pulmonary consumption, Gilbert on, 41
Pulmonary diseases in the United States ........................ 135
Purulent ophthalmia of armies...... 187
Purulent ophthalmia of infants...... 188
Pustular diseases of the scalp ...... 396

R.

Rashness of modern surgeons ...... 170
Rayer on phthisis ................ 562
Reflex action .................... 21
Reform, medical, Sir J. Clark on.... 153
Reform, medical, Dr. Carmichael on 153
Regimen of pregnant women ...... 486
Reparation of bone, King on........ 461
Respiration and animal heat........ 163
Respiration, theory of ............ 369
Retention, spasmodic, treatment of.. 440
Retrospective address, Dr. Streeten's 1
Re-vaccination .................. 11
Rognetta on fistula lachrymalis .... 183
Roux, practice of ................ 560
Roux', M. clinical practice ........ 584
Royal George, wreck of the ........ 633
Rupture of the optic nerve ........ 184

S.

Sampson on criminal jurisprudence.. 79
Sanguineous tumors .............. 228
Scalp, Erichsen on diseases of the .. 379
Scarlatina encephalica ............ 2
Scarlatina, Bright's disease after .... 91
Scarlatina, secondary deposits ...... 249
Scarlet fever .................... 10
Science, unity of.................. 175
Scirrhus of the pancreas .......... 274
Scrivener's spasm ................ 264
Scrofulous ophthalmia ............ 188
Scrofulous ulcer of the neck........ 583
Sedillot on surgical instruction...... 175
Sensation, laws of ................ 17
Sensibility and motion, lesion of .... 586
Serre on the operation for cataract .. 180
Serre on inflammation of the eyes .. 182
Sesquicarb. of iron in traum. tetanus.. 618
Severe wounds, diet in ............ 171
Shapter's climate of Devonshire .... 491
Skin, discoloration of, by nit. of silver 610
Skull, aneurysms within the........ 577
Small-pox, application for pitting in 3
Soden on benzoic acid in urinary disorders ........................ 601
Sounding for stone................ 158
Sounds of the heart .............. 538
Spasm of the thumb, Stromeyer on.. 201
Spermatic cord, affections of the.... 134
Spinal deformities, myotomy in...... 179
Spinal curvature, pathology of...... 516
Spinal curvature, treatment of...... 518
Spine, Harrison on deformities of the 515
Spine, secondary curvatures of the.. 517
Spleen, Jackson on the functions of.. 110
Squamous diseases of the scalp .... 397
Stammering, surgical treatment of .. 198
Stammering, operation for.......... 561
Staphyloma...................... 191
Starched bandage, gangrene from the 600
Statistics of consumption.......... 41
Statistics of the navy ............ 65
Statistics of operations ............ 588
Stomach, perforation of the........ 596
Stone, sounding for .............. 158
Storr's case of hæmorrhage from an abscess ...................... 597
Strabismus ...................... 194
Streeten's retrospective address...... 1
Streeten's topography of Exeter .... 8
Stricture of the urethra............ 118
Stricture, organic lesions from...... 118
Stricture, seat of.................. 120
Stricture, diagnosis of ............ 120
Stricture, treatment of ............ 122
Stricture, on the use of caustic in.... 124
Stricture at the orifice of the urethra 126
Stricture in corpus spongiosum .... 126
Stricture of the membranous portion 127
Stricture, relapse of .............. 128

Stricture and enlarged prostate...... 438
Stricture at the orifice ............ 442
Stricture, division of ............. 445
Strictures of the urethra .......... 436
Stromeyer on spasm of the thumb .. 201
Students and teachers ............ 178
Sugar, detection of, in the urine.. 3, 521
Suicide ........................ 552
Suicide a test of insanity .......... 527
Superficial perineal fascia .......... 158
Supra-ciliary region, wounds of the.. 186
Surgeons, modern, rashness of....... 170
Surgeon's vade mecum, Druitt's .... 520
Surgery, Vidal on ................ 169
Surgery, Cyclopædia of............ 457
Surgical diagnosis ................ 170
Surgical instruction, Sedillot on .... 175
Surgical diseases, influence of constitution on..................... 177
Surgical practice at the Hôtel Dieu .. 560
Sympathetic paralysis ............ 331
Syme's surgical cases and observations 587
Syphilitic ophthalmia.............. 189

T.

Tape-worm, remedy for the ........ 12
Taylor on infanticide.............. 214
Teale's, Mr. case of strangulated hernia 580
Testes, affections of the............ 134
Theory of respiration.............. 369
Therapeutics of children .......... 104
Tic douloureux, Dr. Allnatt on....... 151
Tic douloureux, causes of....... ... 152
Tic douloureux, its treatment ...... 152
Tinea, treatment of................ 563
Tobacco, influence of, on phthisis .. 541
Torquay, description of............ 494
Torsion of arteries................ 172
Transactions of Provincial Association 1
Traumatic tetanus, sesquicarbonate of iron in......... ............... 618
Treatment of tinea................ 563
Trichiasis ...................... 185
Tubarian gestation, Dr. Glover's case, 615
Tuberculous degenerescence ........ 465
Tumors of the bony pelvis.......... 219
Tumors of the uterus and vagina.... 221
Typhoid fever, Baron Michel on .... 165

U.

United States, Forry on the climate of 135
United States, pulmonary diseases in 135
Unity of Science ................ 175
Urethra, functional diseases of the .. 117
Urethra, stricture of the .......... 118
Urethra, obstructions of, from injury, 447
Urethra, strictures of the.......... 436
Urethro-prostatic discharges........ 411
Urinary abscess .................. 131
Urinary fistulæ .................. 133
Urinary bladder, anatomy of the .... 157
Urinary concretions, Bird on........ 232
Urinary organs, Brodie on the ...... 401
Urinary disorders, benzoic acid in.... 601
Urine, detection of sugar in the .... 3
Urine, a test of pregnancy ... .... 4
Urine, infiltration of .............. 130
Urine, stagnation of ..... ........ 419
Urine, retention of....... ........ 421
Urine, incontinence of........ 423, 450
Uteri carcinoma.................. 225
Uterus, injections into the ........ 4
Uterus, tumors of the ............ 221
Uterus, abscess of the ............ 222
Uterus, fibrous tumors of the ...... 224

V.

Vaccination in France ............ 205
Vade mecum, Druitt's ............ 520
Vagina, tumors of the ............ 221
Vagina, abscess of the ............ 227
Vagina, polypus of the............ 227
Vagina, malignant tumors of the.... 228
Varices at the neck of the bladder .. 412
Variolæ vaccinæ, Ceely on the...... 13
Variolæ vaccinæ, ulcerative stage of.. 15
Vegetable substances, component materials of ...................... 161
Venous hæmorrhage from an abscess 597
Veru montanum, affections of the .. 412
Vesiculæ seminales, diseases of the .. 405
Vesicular diseases of the scalp ...... 385
Vidal on surgery ................ 169
Vivisections, M. Longet's .......... 536
Voluntary motion, laws of.......... 17

W.

Water-cure, Dr. Wilson on the...... 156
Watson on polypous tumors of the nose.......................... 252
Weatherhead on gout.............. 509
Webster on the admission of pupils to Bethlem ...................... 147
Wilson on the water-cure .......... 156
Wound of the optic nerve.......... 184
Wound, penetrating, of both lungs .. 614
Wounds, influence of external circumstances on ...... .............. 177

### BIBLIOGRAPHICAL RECORD.

1. The Naturalist's Library. Ornithology. Vol. XII. Birds of Great Britain, &c. By Sir W. Jardine, Bart. Edinb. W. B. Lizars. 1842.

---

2. Elements of General Pathology. By the late Dr. J. Fletcher. Edited by Dr. J. J. Drysdale, and Dr. J. R. Russell. Octavo, pp. 520. Maclachlan, Stewart, and Co. Edinburgh, 1842.

---

3. The Medical Times, for the Quarter ending 30th March, 1842. *In exchange.*

☞ *There is a prodigious improvement in the manner and matter of this our contemporary, since the late editorial change.*

---

4. Annales de la Chirurgie Francaise et Etrangere. Par MM. Begin, Marchal, Velpeau, &c. No. 18. Mars, 1842. Bailliere, Regent Street.

---

5. Pharmaceutical Journal and Transactions. Edited by Jacob Bell. No. X. April, 1842. *In exchange.*

---

6. Traité Pratique sur les Maladies des Organs Genito-Urinaires. Par le Docteur Civiale. Premiere Partie. Maladies de l'Urethre—avec trois Planches. Paris, 1837.

---

7. Traité Pratique sur les Maladies des Organs Genito-Urinaires. Deuxieme Partie, Maladies du Col de la Vessie et de la Prostate, avec cinque Planches. Paris, 1841.

---

8. Traité Pratique sur les Maladies des Organs Genito-Urinaires. Troisieme Partie. Maladies du Corps de la Vessie. Paris, 1842.

---

9. Guy's Hospital Reports. No. XIV. April, 1842. By Dr. Barlow and Dr. J. Babington. Highley, 1842.

---

10. Discourse on the enlarged and pendulous Abdomen, &c. &c. Second Edition, with a Dissertation on Gout. By Richard Frankum, Esq. Duodecimo, pp. 121. Longman and Co. 1842.

---

11. Researches into the Natural History of Mankind. By J. C. Prichard, M.D. Third Edition, Vol. III. Part I. Researches into the Ethnography of Europe, with Plates. Octavo, pp. 506. Sherwood and Co. 1841.

---

12. A Practical Treatise on Auscultation. By M. Barth, M.D. Translated, with Notes, by Patrick Newbigging, M.D. Octavo, pp. 398. Maclachlan and Co. 1842.

---

13. On the Cure of Bronchitis, Consumption, &c. &c. by Inhalation. By Edw. Jenner Coxe, M.D. Duodecimo. Philadelphia, J. Dobson. 1841.

---

14. Preservation of the Teeth indispensable to Comfort, Appearance, Health, and Longevity; being a Second Edition of Dental Practice. By J. Gray, Dentist, 25, Old Burlington Street. Duodecimo, 1838.

---

15. Practical Treatise on Stammering. By Joseph Poett, M.R.C.S. 5th Edition. Highley, 1842.

---

16. De la Menstruation, considerée dans ses Rapports Physiologiques et Pathologiques. Par E. Brierre de Boissement, &c. Paris, Bailliere, 1842.

---

17. On the Mutual Relation between Anatomy, Physiology, Pathology, and Therapeutics, and the Practice of Medicine. Being the Gulstonian Lectures for 1842. By Marshall Hall, M.D. Illustrated by three engraved plates. H. Bailliere, Regent Street, 1842.

---

18. The Elements of Materia Medica and Therapeutics. By Jonathan Pereira, M.D. F.R.S. &c. Two vols. Octavo, pp. 1700. Longmans, 1842. Second Edition, enlarged.

---

19. The Cyclopædia of Anatomy and Physiology. Edited by Robert Todd, M.D. F.R.S. &c. Part XXIII. Containing Monotremata, (R. Owen, Esq.) Motion, (J. Bishop, Esq.) Mucus, (Dr. G. O. Rees.)

Mucous Membrane, (W. Bowman, Esq.) Illustrated with numerous Engravings. London, Sherwood and Co. April, 1842. Price 5s.

---

20. A Series of Anatomical Plates, in Lithography, &c. &c. By JONES QUAIN, M.D. &c. Division I. Muscles. Fasciculus 99. Price 2s. J. Taylor, London.

---

21. The Anatomy of the Arteries of the Human Body, with its Application to Pathology and Operative Surgery. In Lithographic Drawings, with Practical Commentaries. The Delineations by Jos. MACLISE, Esq. Surgeon. Part XI. Price 12s. Taylor, London. 1842.

---

22. Text-book of Anatomy, for Junior Students. By ALEX. JARDINE LIZARS, M.D. &c. Part II. Containing the Senses, Brain, and Nerves. Bell and Bradfute, Ed. S. Highley, London. Small 8vo.

☞ *Part* III. *will complete the work.*

---

23. The Education of Mothers of Families; or, the Civilization of the Human Race by Women. By AIME-MARTIN, &c. Translated from the Third French Edition, by EDWIN LEE, Esq. Member of the principal European Medical and Chirurgical Societies, &c. Octavo, pp. 384. Whittaker and Co. London, 1842.

---

24. An Inquiry into the Nature and Causes of Epilepsy; with the Function of the Spleen, and the Use of the Thyroid Body, &c. By JOHN JACKSON, M.R.C.S. London, 8vo. pp. 107. Whittaker, London. 1842.

---

25. Statistical Tables of the Royal Infirmary of Edinburgh, &c. By Professor REID, of St. Andrews, lately Superintendant of the Royal Infirmary.

☞ *These consist of* 20 *numerical tables very valuable, but incapable of analysis.*

---

26. The Nervous System and its Functions. By HERBERT MAYO, F.R.S. &c. Octavo, pp. 182. Parker, West Strand. 1842.

---

27. The Climate of the United States, and its Endemic Influences, based chiefly on the Records of the Medical Department, and Adjutant-General's Office, United States' Army. By SAMUEL FORRY, M.D. Pp. 380. New York, 1842.

28. The Mineral Springs of Aix-la-Chapelle and Bourcette; with some Account of the Curiosities of both Places and the Environs. By L. Wetzlar, M.D. Physician at Aix-la-Chapelle. Octavo, pp. 88. Churchill, 1842.

---

29. On the Theory and Practice of Midwifery. By FLEETWOOD CHURCHILL, M.D. Physician to the Western Lying-in Hospital, &c. Octavo, pp. 477, (very small type,) illustrated by upwards of one hundred highly finished wood-engravings by Bagg. Renshaw, London. 1842.

---

30. Dr. Hooper's Physician's Vade Mecum; or Manual of the Principles and Practice of Physic. New Edition, considerably enlarged and improved, with an Outline of General Pathology and Therapeutics. By WILLIAM AUGUSTUS GUY, M.D. of King's College, London. Octavo, pp. 493. Renshaw and others, London, 1842.

---

31. The Anatomist's Vade Mecum: a System of Human Anatomy. By ERASMUS WILSON, with 167 Illustrations by Bragg. Second Edition, pp. 596. Churchill, London, 1842.

---

32. Dr. Tavernier's Treatise on the Treatment of Deformities of the Spine by the Lever-belt with Inclination Busk; without Extension Beds or Crutches, &c. Translated into English, with Critical Analysis, &c. By W. BREWER, M.D. 8vo. (double columns), pp. 43. Simpkin and Co. 1842.

---

33. Pharmaceutical Journal and Transactions, No. 12. June 1, 1842.

---

34. Atlas illustrating the Anatomy of the Human Body. By J. CRUVEILHIER, M.D. The figures by Em. Beau, with Explanations by C. Bonamy, M.D. Part 8. Bailliere, Regent Street. 1842.

---

35. Provincial Medical Journal, &c. *In exchange.*

---

36. A History of British Birds. By W. YARRELL, F.L.S. &c. Parts 29, 30, 31. London, J. Van Voorst, Paternoster-row. Price 2s. 6d. each.

---

37. Tic Douloureux; or Neuralgia Facialis, and other Nervous Affections; their Seat, Nature, and Cause, with Cases, illustrating successful Methods of Treatment.

By R. H. Allnatt, M.D. A.M. F.S.A. Octavo, pp. 1842. Churchill, 1842.

38. Solution du Probleme de la Population et de la Subsistence, soumise a un Medecin dans une Series de Lettres. Par Charles Loudon, M.D. &c. Paris, 1841.

39. Observations addressed to the President elect of the British Association, &c. By Alex. Nasmyth, M.R.C.S. F.L.S. &c. Octavo, pp. 58. 1842.

40. A Practical Treatise on the Cure of Diseases by Water; being a New Mode of restoring Injured Constitutions, &c. By James Wilson, M.D. Octavo, pp. 202. Churchill, 1842.

41. Twelfth Annual Report of the Belfast District Lunatic Asylum, March, 1842.

42. La Clinique des Hopitaux des Enfans, &c. &c. Avril, 1842.

43. Remarks on Medical Reform, in a Letter to Sir James Graham, Bart. By Sir James Clark, M.D. Bart. Pp. 30. 20th June, 1842.

### TO CORRESPONDENTS, &c.

### SOCIETY FOR IMPROVING THE CONDITION OF THE INSANE.

This Society has been formed under distinguished auspices, lay and professional, and for the most laudable purposes. This Society proposes to offer premiums, for the present year, as follows:—

1. To the Author of the best essay on the arrangement and nomenclature of mental disorders, a premium of 20 Guineas.
2. To the male attendant who shall produce the best testimonials, a premium of 5 Guineas.
3. To the female attendant who shall produce the best testimonials, a premium of 5 Guineas.

The essays and testimonials to be addressed to Mr. T. C. Morison, 397, Oxford Street, London, Honorary Secretary, (pro tempore,) of the Society for improving the condition of the Insane, on or before the 1st day of November, 1842.

### DR. MONTGOMERY ON PREGNANCY.

We perceive with satisfaction that the favorable opinion expressed of Dr. Montgomery's work in this Journal (Jan. 1838) has been amply confirmed by the general assent of the profession and public, not only in these countries, but on the Continent and in America.

It has been translated into German at Bonn at the recommendation of Professor Kilian, who has added an introductory chapter, and thereby given a practical proof of the high estimation in which the book is held by so competent a judge; it has also been re-printed in America, and we are informed that the re-print there is already exhausted, and that it is the highest charged book in the States.

Mr. Roberts, Assistant-Surgeon 59th, and many other Correspondents, are thanked for their communications; but they will perceive a notice in the last number of the Journal that the Editors have determined not to insert original communications in future.

